# Miniaturized protein profiling permits targeted signaling pathway analysis in individual circulating tumor cells to improve personalized treatment

**DOI:** 10.1186/s12967-024-05616-7

**Published:** 2024-09-20

**Authors:** Mahdi Rivandi, André Franken, Liwen Yang, Anna Abramova, Nadia Stamm, Jens Eberhardt, Berthold Gierke, Meike Beer, Tanja Fehm, Dieter Niederacher, Michael Pawlak, Hans Neubauer

**Affiliations:** 1 Department of Obstetrics and Gynecology, University Hospital and Medical Faculty of Heinrich Heine University Duesseldorf, Duesseldorf, Germany; 2 Center for Integrated Oncology (CIO Aachen, Bonn, Cologne, Duesseldorf), Duesseldorf, Germany; 3 Sartorius Automated Lab Solutions GmbH, Jena, Germany; 4 NMI TT GmbH, Protein Profiling, Reutlingen, Germany; 5 NMI Natural and Medical Sciences Institute at the University of Tuebingen, Reutlingen, Germany

**Keywords:** Circulating tumor cell, Breast cancer, Protein analysis, Single cell proteomics, Personalized medicine

## Abstract

**Background:**

Traditional genomic profiling and mutation analysis of single cells like Circulating Tumor Cells (CTCs) fails to capture post-translational and functional alterations of proteins, often leading to limited treatment efficacy. To overcome this gap, we developed a miniaturized ‘protein analysis on the single cell level’ workflow—baptized ZeptoCTC. It integrates established technologies for single-cell isolation with sensitive Reverse Phase Protein Array (RPPA) analysis, thus enabling the comprehensive assessment of multiple protein expression and activation in individual CTCs.

**Methods:**

The ZeptoCTC workflow involves several critical steps. Firstly, individual cells are labeled and isolated. This is followed by cell lysis and the printing of true single cell lysate preparations onto a ZeptoChip using a modified micromanipulator, CellCelector™. The printed lysates then undergo fluorescence immunoassay RPPA protein detection using a ZeptoReader. Finally, signal quantification is carried out with Image J software, ensuring precise measurement of multiple protein levels.

**Results:**

The efficacy of ZeptoCTC was demonstrated through various applications. Initially, it was used for measuring EpCAM protein expression, a standard marker for CTC detection, revealing higher levels in single MCF-7 over MDA-MB-231 tumor cells. Furthermore, in Capivasertib (Akt-inhibitor)-treated MCF-7 single cells, ZeptoCTC detected a 2-fold increase in the pAkt/Akt ratio compared to control cells, and confirmed co-performed bulk-cell western blot analysis results. Notably, when applied to individual CTCs from metastasized breast cancer patients, ZeptoCTC revealed significant differences in protein activation levels, particularly in measured pAkt and pErk levels, compared to patient-matched WBCs. Moreover, it successfully differentiated between CTCs from patients with different Akt1 genotypes, highlighting its potential to determine the activation status of druggable cancer driving proteins for individual and targeted treatment decision making.

**Conclusions:**

The ZeptoCTC workflow represents a valuable tool in single cell cancer research, crucial for personalized medicine. It permits detailed analysis of key proteins and their activation status of targeted, cancer-driven signaling pathways in single cell samples, aiding in understanding tumor response, progression, and treatment efficacy beyond bulk analysis. The method significantly advances clinical investigations in cancer, improving treatment precision and effectiveness. The workflow will be applicable to protein analysis on other types of single cells like relevant in stem cell, neuropathology and hemopoietic cell research.

**Supplementary Information:**

The online version contains supplementary material available at 10.1186/s12967-024-05616-7.

## Background

In recent years, cancer treatment has advanced with targeted therapies, reducing side effects and improving patient survival. However, acquired resistance in tumor cells necessitates more accurate therapy prediction methods. Traditional biopsies often fail to capture the disease’s current state due to limitations in time and space [1].

Liquid biopsies, particularly focusing on circulating tumor cells (CTCs), offer a dynamic, non-invasive alternative. Elevated CTC counts correlate with lower survival rates in various cancers, highlighting their potential as prognostic biomarkers [2–6]. For instance, the presence of five or more CTCs in 7.5 mL of blood is linked to worse outcomes in metastasized breast cancer (MBC) [7]. Beyond enumeration, the genotypic and phenotypic analysis of CTCs is gaining traction, offering insights into tumor biology and potential treatment strategies through multi-parametric analyses [3, 8]. Studies like the STIC-CTC and DETECT-III trials emphasize the predictive value of CTC quantity and characteristics [4–6] and genomic analysis of CTCs has revealed mutations in genes such as AKT1 and PIK3CA, crucial for targeted breast cancer treatments [9–11]. Drugs targeting these mutations, like Alpelisib and Capivasertib, provide new treatment possibilities [12–14]. However, the relationship between established hotspot mutations and dynamic protein activities is complex, influenced by factors like additional mutations and posttranslational modifications. This complexity underscores the need for protein analysis at the single-cell level to understand the functional impact of mutations [15–19].

Current protein analysis technologies face limitations in detection sensitivity and high cell number requirements, making them inadequate for single CTC analysis [20]. Techniques such as flow cytometry, mass spectrometry, and mass cytometry—CyTOF, though advanced, are not optimized for single-cell input and often suffer from analyte loss [21–28].

To address these challenges, common Reverse Phase Protein Arrays (RPPA) have been developed as a sensitive proteomic immunoassay. RPPA, derived from classical western blot, allows for high-throughput, minute sample consumption, and focused profiling of signaling proteins in small sample sizes [25, 26]. The Zeptosens RPPA system, combined with planar waveguide technology, enables the detection of proteins in low zeptomole ranges, possibly from single cells but has not yet been set-up and tested for the detection of proteins from true single cell sample preparations. [29–31].

Our further miniaturized, single-cell protein profiling approach combines the ultra-sensitive Zeptosens fluorescence detection RPPA system with a robust workflow for isolation, preparation and quantitative detection at the single cell level and enables multiple protein analysis of individual tumor cells and CTCs as demonstrated. The method involves (a) the use of the pre-analytical, FDA-approved CellSearch® system (Menarini Silicon Biosystems, Bologna, Italy) to identify and isolate patient CTC populations based on their expression of the epithelial cell adhesion molecule (EpCAM) and cytokeratins (CK) and the absence of the hematopoietic cell surface protein CD45, followed by (b) the single CTC isolation, single CTC lysis, and subsequent spotting of the single CTC lysate preparations onto hydrophobic ZeptoChips using the state-of-the-art automated micromanipulator CellCelector™ (Sartorius, Jena) [32]. As a last step (c) ZeptoCTC is applying adapted Zeptosens RPPA to process the printed single cell lysate sample arrays, measure multiple protein analyte fluorescence assays, and finally determine and relate quantitative signal levels of total and phosphorylated forms of the measured proteins in the printed samples and controls. We further demonstrate the clinical applicability of this highly sensitive and robust workflow for single-cell protein analysis by determining treatment-relevant relative expression of total and phosphorylated Akt and Erk1/2 signaling proteins in individual CTCs of MBC patients.

## Methods

### Cell lines

Breast cancer cells from cell lines MDA-MB-231 and MCF-7 were used (ATCC, Manassas, United States; catalog numbers: MDA-MB-231: HTB-26, and MCF-7: HTB-22) and cultured in RPMI 1640 containing 10% fetal calf serum and 1% Penicillin-Streptomycin (all Thermo Fisher Scientific) and routinely maintained in humidified atmosphere of 5% CO2 and 95% air at 37 °C. Twenty-four hours before treatment with Capivasertib the culture medium of MCF-7 was replaced by phenol red-free RPMI supplemented with 5% charcoal-stripped FCS. Half of the synchronized cells were then grown in the above-described medium supplemented with 5 µM Capivasertib, the other half was cultured as before. Cells were negatively tested for mycoplasma.

### Clinical samples

Diagnostic Leukapheresis (DLA) was performed on MBC patients at the Department of Obstetrics and Gynecology of University Hospital Duesseldorf, following an approved protocol [33, 34]. Written informed consent was obtained from the patients, and approved by the Ethics Committee of the Medical Faculty of Duesseldorf (Ref-No: 3460) and all procedures were conducted in accordance with the principles outlined in the Helsinki Declaration. Subsequently, DLA samples were cryopreserved and thawed using methods described in a prior study [35].

### Reagents

For single cell lysis, Zeptosens cell lysis buffer CLB1 (50 mL, Zeptosens #9000) and Zeptosens print buffer CSBL1 (50 mL, Zeptosens #9020) were utilized [29]. The Micro pick 48® slide was coated with a solution comprising 2% bovine serum albumin (BSA) in Dulbecco’s phosphate-buffered saline (DPBS). This coating solution was applied to the slide to prevent protein loss during the workflow of printing lysate on the ZeptoChip slide.

### Western blot analysis

RIPA buffer (50 mM TRIS; Sigma-Aldrich, T1503) was used to lyse the cells. After measuring protein concentration with the BCA assay (Thermo Fisher Scientific, 23225), 30 µg of total protein lysate was loaded for each sample onto Mini-PROTEAN® Precast Gels (Bio-Rad, 4568123) with 4 × Laemmli buffer (Bio-Rad, Feldkirchen, Germany, 1610747). Separated proteins were transferred to Immun-Blot® PVDF Membranes (Bio-Rad Laboratories, Inc., Hercules, California). After blocking the membrane with 1% BSA in TBS and 0.1% Tween (Sigma-Aldrich, St. Louis, Missouri) (TBS-T) for 60 min at room temperature, the following antibodies were used: anti-Akt (pan) (Cell Signaling Technology, 4691) and anti-phospho-Akt (Ser473) (Cell Signaling Technology, 4060). The membranes were incubated overnight at 4 °C. After washing the membranes, they were visualized with horseradish peroxidase-conjugated anti-rabbit IgG (Cell Signaling Technology) at a 1:2000 dilution. Data were analyzed using Image Lab 2.0.1 software and normalized to β-actin expression.

### RPPA

The current RPPA protein analysis was based and further developed on RPPA-established standard routines, equipment, consumables, and reagents using Zeptosens technology as described previously [27] and shortly outlined here. All new RPPA adaptation steps are described separately in the Results section. For standard RPPA, lysates are routinely produced by strong denaturing lysis in CLB1 from bulk cell or tissue sample material. For array printing, the lysates are subsequently diluted ten-fold in CSBL1 to reach final print concentrations of typically 0.2 ug/µL total protein, and printed onto hydrophobic ZeptoChips (6 pre-defined array fields per chip) using routinely a piezo-electric non-contact NanoPlotter2 (GeSiM, Grosserkmannsdorf Germany) with the print samples provided in standard 384-well plates, and printing performed by single droplet deposition (0.4 nL per spot) in duplicate spots per lysate sample. In this workflow, the whole lysis and printing routine has been newly developed and was transferred to a capillary-based, single cell picking device (CellCelector™ micromanipulator, Sartorius, Jena Germany), modified for in situ lysis and printing as described in Results. Standard cell lysates are routinely co-printed into standard RPPA (MCF-7 treated (activated) and control cell lysates, at 0.2 µg/µL protein print concentration in CLB1/CSBL), and were also applied and co-printed into the new single cell sample arrays, as quality control and benchmark. Finally, printed ZeptoChip arrays are blocked with 3% w/v albumin, washed in distilled water, dried, and stored at 4 °C in the dark until use.

#### Protein array immunoassays

Protein signals were measured in a direct two-step sequential immunoassay using a sensitive and quantitative fluorescence read-out. A single array was probed for each protein marker of interest. The primary antibodies were selected from the current established list of specific RPPA assays (currently 700+). The antibodies were up-front well-characterized for analyte specificity and verified extensively in other studies with various sources of cell and tissue samples. For each protein assay, the primary antibody at respective dilution was incubated in Zeptosens assay buffer overnight (19 h) at room temperature, arrays were washed once in assay buffer and incubated for 45 min with Alexa647-labeled anti-species secondary antibody (Invitrogen, Paisley, UK). Arrays were then washed and imaged in solution using a ZeptoREADER instrument (Zeptosens) using the red laser. Typically, six fluorescence images are recorded for each array at exposure times of between 0.5 and 16 s, including negative control assays in the absence of primary antibody (blank assays) to measure non-specific signal contributions of the secondary antibody reagents. All primary antibodies applied were from Cell Signaling Technology Inc. (Danvers MA, USA): CST 3599 for EpCAM, CST4685 for Akt, CST 4060 for Akt-P-S473, CST4695 for Erk1/2, CST 9101 for Erk1/2-P-Thr202/Tyr204.

#### Image capture and analysis

For each array (primary antibody and blank assays), the image taken at the longest exposure time without showing any saturation was analyzed using ImageJ software as described in Results in more detail. The mean fluorescence intensity (MFI) of each sample was determined from analyzed mean single spot signals, averaged over the number of blank-corrected, replicate mean single spot signals (typically *n* = 3). Coefficients-of-variation (CVs) were calculated as the ratios of the respective standard deviations (shown as error bars in the graphs) and MFIs.

### Data analysis

Fluorescence signals from the printed spots were analyzed using standard Image J software routines (version 1.50f, NIH, USA). Spot signals and mean fluorescence intensities (MFIs) were subjected to statistical analysis using p-values from paired t-tests. The data analysis was performed using Excel software.

## Results

### The ZeptoCTC workflow

**Fig. 1:**
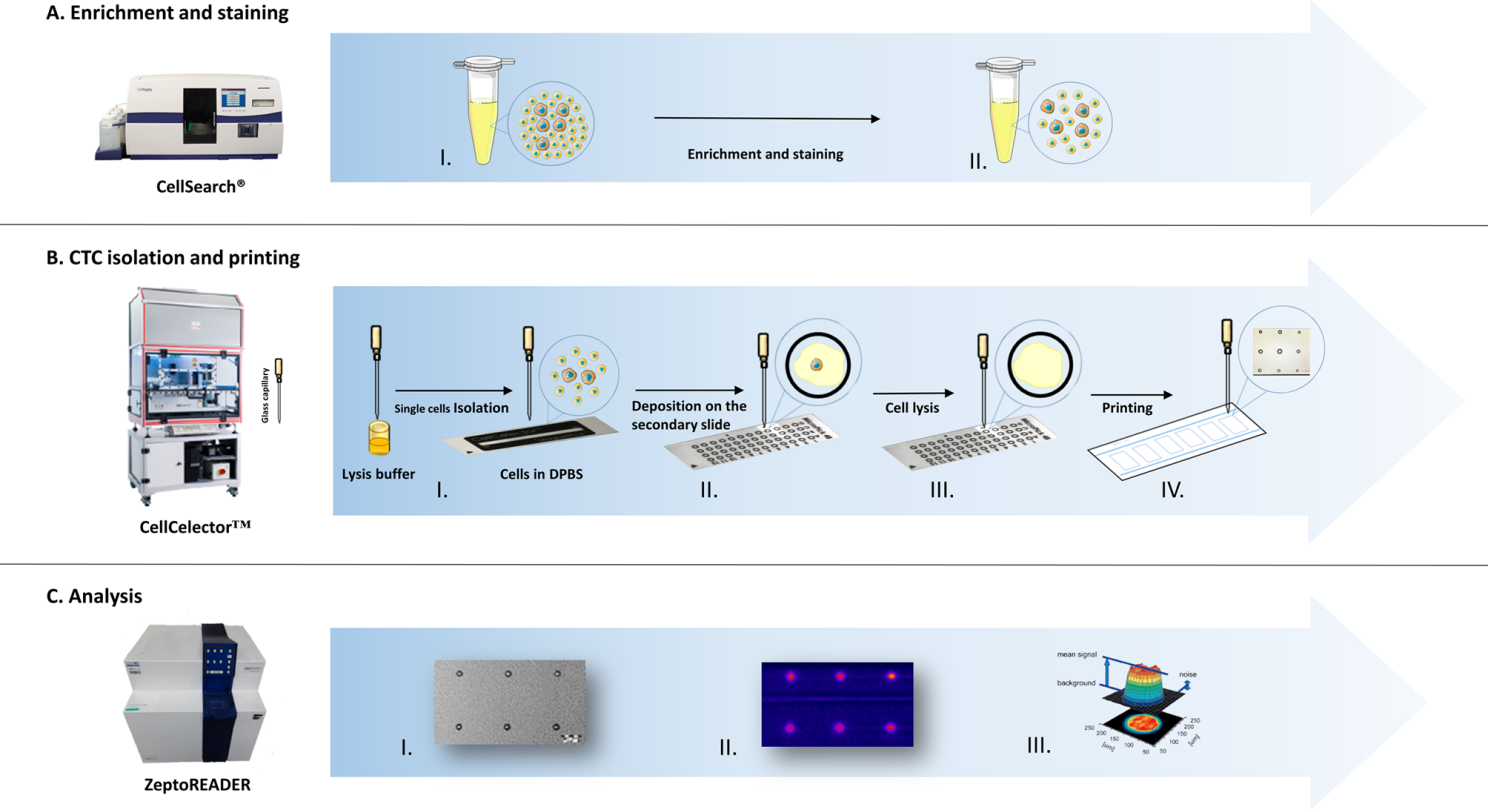
Overview of the established workflow. Depicted is the established workflow for isolating and analyzing single CTCs with Reverse Phase Protein Arrays (RPPA). The workflow consists of three primary steps: (**A**) Capture and verification of CTCs using Diagnostic Leukapheresis (DLA) combined with the CellSearch® system. (**B**) Single cell/CTC picking with a glass capillary, followed by in situ lysing and mixing on a Micropick 48 slide™ and subsequent printing onto ZeptoChip using a modified CellCelector™ instrument. (**C**) RPPA analysis of the chip using the ZeptoReader

The concept for setting up a true single cell protein analysis workflow was to combine and fully integrate already existing, well-validated sub-workflows of selected mature technology modules (schematically displayed in Fig. 1). This includes using the DLA and CellSearch® system to provide well-characterized CTC preparations (A), the CellCelector™ automated micro-manipulation system to isolate single cells by capillary picking from cell culture vessels, to prepare true single-cell lysates and to print in situ single cell lysates in array format (B), and the Zeptosens RPPA module for sensitive and robust downstream protein profiling, including the usage of the adapted ZeptoChip platform, matched lysis, and printing buffer reagents, upfront validated specific assay antibodies and customized single cell sample array and data analysis routines (C). The modules of the workflow concept were stepwise realized, adapted, and tested with single cells from tumor cell line cultures and then adjusted afterward to CTC.

In panel A, the CTCs are captured using the CellSearch® Profile Kit, and surface markers are stained for imaging. In panel B, a single cell with approximately 20 nL of DPBS is aspirated from a culture dish into the lysis buffer pre-filled glass capillary of the CellCelector™ instrument (total inner volume 40 nL). The CellCelector™ is programmed to deposit 2 nL volume of single cell lysate per sample spot in an array, therefore a total of 40 nL sample lysate volume is sufficient to produce up to 6 sample arrays per chip with 3 technical replicate spots per sample per array. Finally, in panel C, downstream RPPA analysis using the ZeptoReader: immunoassays for selected protein markers are performed on the printed and blocked ZeptoChip lysate arrays, fluorescence images of the arrays are recorded and mean spot assay signals quantified with ImageJ software.

It is worth noting that the miniaturization described in this workflow represents a significant improvement over existing standard RPPA techniques. While some RPPA methods, such as Zeptosens RPPA, have demonstrated single-cell sensitivity, they still rely on standard vial 96/384 microtiter plate formats and printing robot routines that require large volumes of buffers with many µL of dead volumes, typically necessitating high numbers of starting cancer cells (on the order of 10^4^ to 10^6^). The main challenge of miniaturizing this approach was to achieve comparably good protein readout performance from true single (or few) cell preparations while ensuring all key specifications for each of the involved technology modules, such as lysis and printing.

We performed multiple replicates of lysates obtained from individual cells during the experiments and deposited them onto a chip. This approach enabled us to employ both blank and target protein assays to measure and quantify the signals, as described in their respective sections or figures. The average fluorescence signals from the printed spots were analyzed using standard Image J software routines (version 1.50f, NIH, USA) and subjected to statistical analysis using a paired t-test performed with Excel software.

#### Optimizing single cell lysis

A critical step in the workflow was to compose a specific mixture of cell lysis buffer and print buffer to meet the key requirements of this miniaturized one step approach: high efficiency for full cell lysis and good printability from the same composition in parallel; procedures, which for regular RPPA are so far being executed as separate steps (see [31] for more details on buffer requirements). Based on the experience with Zeptosens RPPA lysis buffer CLB1 and print buffer CSBL1, we followed the approach of presenting a concentrated mixture of CLB1:CSBL1 pre-aspirated into the glass capillary and subsequently picking the single tumor cell accompanied with a controlled shot of PBS, which dilutes out the CLB1/CSBL1 mixture. CLB1 has a strong denaturing activity compared to CSBL1 due to its higher urea and thiourea concentrations (7 M urea, 2 M thiourea, and high detergent in CLB1, 3.89 M urea, 1.11 M thiourea in CSBL1, no detergent). On the other hand, CSBL1 containing 11.1% glycerol is beneficial for good printing behavior of the cell lysate and has a low tendency to crystallize. The final buffer composition in a total volume of 40 nL had to guarantee efficient and full lysis at a short duration with high protein yield, prevent crystallization in the glass capillary, and ensure good compatibility with the array printing and final RPPA analysis process. To this aim, both buffers as well as different mixtures of both were selected and tested.

**Fig. 2:**
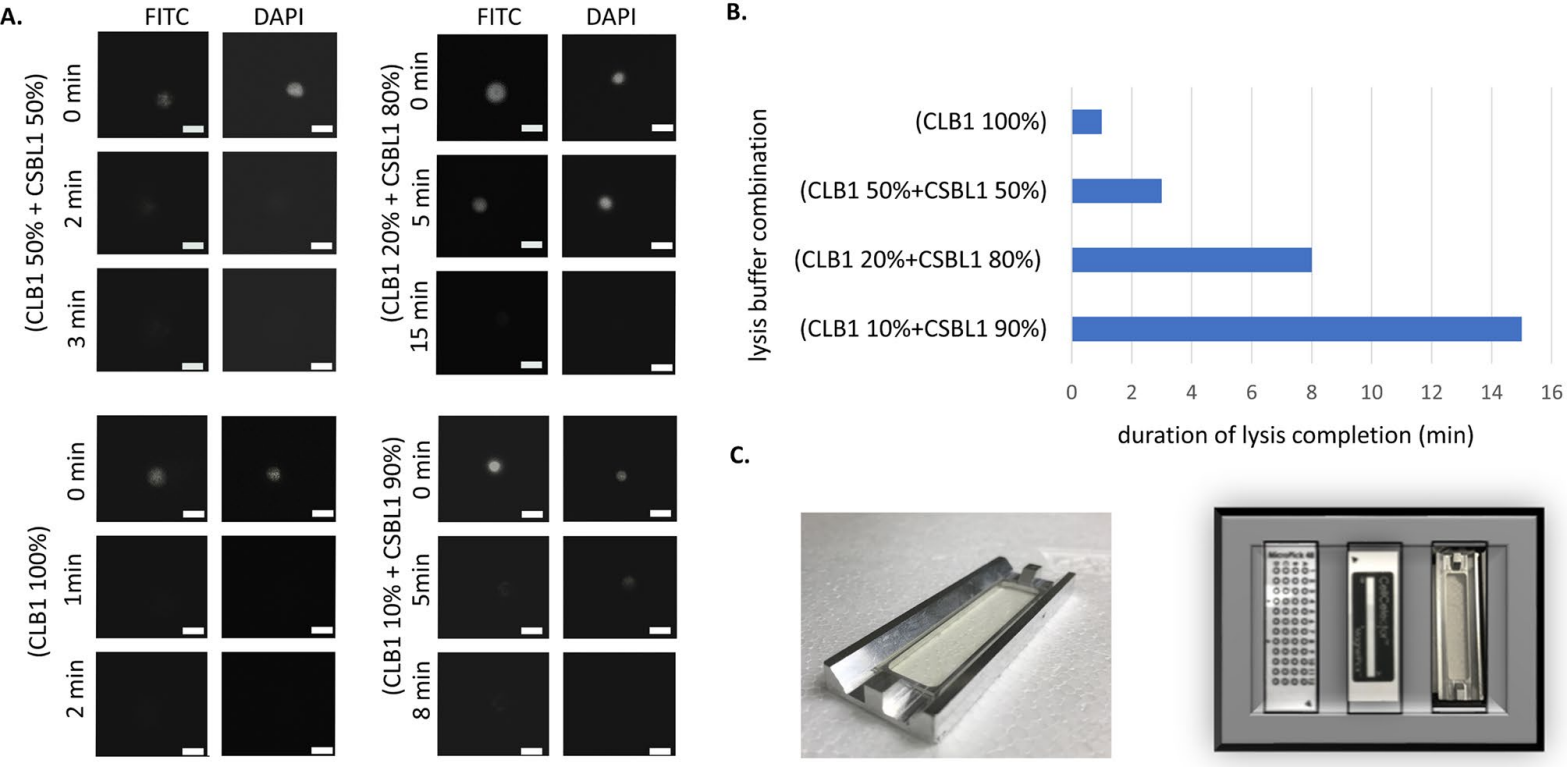
Optimization of conditions and hardware for single cell lysis and manipulation. (**A**) Monitoring of single cell lysis efficiency upon variation of lysis buffer composition. For better visualization, MCF-7 cells were prelabeled with Celltracker Green and NucBlue™ Live ReadyProbes™, and fluorescence images under the microscope were taken at indicated time points during lysis; Scale bar: 25 μm, Magnification:10x; DAPI (nucleus) and FITC (cytoplasm); (**B**) Lysis efficiency: comparison of complete lysis durations (min) for different CLB1:CSBL1 ratios; (**C**) Design of the holder for the ZeptoChip (left) and the adapter for the Micropick 48® slide, the Magnetpick® slide, and for the ZeptoChip (plus its holder) to position them precisely onto the CellCelector’s microscope stage (right)

First, we determined completion of cell lysis indicated by changes in the cell’s and nucleus’ morphologies by microscopy in a time-lapse experiment with single cells of cultured MCF-7 breast cancer cell line, which were prelabeled with Celltracker Green CMFDA (Life Technologies, #C2925); final concentration 5 µM (1:2000) and NucBlue™ Live ReadyProbes™ Reagent (Hoechst 33342) (Invitrogen, R37605) in FITC and DAPI and incubated with different CLB1:CSBL1 ratios (1:0, 1:2, 1:5 and 1:10) (Fig. 2A). All CLB1:CSBL1 ratios lysed the cells completely in between 1 and 16 min. The fastest cell lysis with approx. 1 min was observed with CLB1-only buffer, followed by the 1:1 mix of CLB1 and CSBL1 with approx. 3 min. Lysis buffer ratios with higher contents of CSBL1 buffer resulted in lysis durations between 7 and 16 min (Fig. 2B). After careful consideration, we selected the 1:1 mix of CLB1 and CSBL1, which contained less (50%) detergent than CLB1-only buffer and caused minimal crystallization in the micromanipulator’s capillary. Although CLB1 buffer alone lysed the cells faster than the 1:1 mix of CLB1 and CSBL1, crystallization in the capillary made it unsuitable for our purposes.

In order to optimize single cell lysis and manipulation, additional hardware and software settings were implemented and adjusted. An adaptor was designed and implemented to position the ZeptoChip on the CellCelector™ microscope stage for fast and precise spotting of cell lysates. This setting was highly important to avoid drying artifacts due to the handling of the miniature volumes of single cell lysate (Fig. 2C).

Consequently, we finally applied this setting together with CellCelector™ glass capillaries pre-filled with 20 nL of a 1:1 mixture CLB1:CSBL1 and single cells picked with another 20 nL excess volume of DPBS under CellCelector™ control for all subsequent experiments.

#### Single cell lysate printing

To reproducibly print well-mixed single cell lysates with the CellCelector™, a series of steps were taken. Pre-labeled MCF-7 cells were scanned and lysed as described above. Although the lysis procedure immediately started in the micromanipulator’s glass capillary indicating that cell lysis buffer and DPBS were mixed, complete cell lysis and homogenous mixing of all components must be guaranteed. To this aim a total 40 nL of whole single cell lysate was released from the glass capillary into a circled hydrophilic area of a Micropick 48™ slide. The release of the single cell lysate mixture forced instantaneous good mixing of the glass capillary’s content; the completion of cell lysis was video-tracked under the microscope. After a final, efficient mixing by 5 times aspiration-and-release with the glass capillary on the Micropick 48™ slide, the printing procedure of the cell lysate sample started immediately using the same glass capillary and a ZeptoChip adjacently mounted on the CellCelector™ deck (Fig. 2C). Technical replicates of single droplet spots (typically *n* = 3) for each sample were printed by aspirating and release of approximately 2 nL volume portions of the complete 40 nL single cell lysate on the ZeptoChip surface, moving the glass capillary to pre-defined spot positions of the 6 pre-defined array fields of the ZeptoChip, one after the other. The lysate volume portions were deposited in close contact with the solution and the ZeptoChip surface. Up to three technical replicate spots per single cell sample were printed as inline series onto each of the 6 array replicate fields per ZeptoChip to examine the reproducibility of printing (see additional file 1).

The entire printing process was executed in a controlled atmosphere at 65% humidity. The printed ZeptoChip was kept at RT for 24 h followed by a drying incubation for 3.5 h at 37 °C. Finally, ZeptoChip was stored in the dark at 4 °C until further use.

#### Single cell protein analysis

The main difference to conventional RPPA is the way of producing the arrays, i.e., we printed them directly in situ after the single cell lysate preparation avoiding dead volumes. The following steps, especially performing the protein assays on the blocked and printed chips were conducted similarly as previously reported [31].

The available array replicates per chip were used to measure in parallel the expression and phosphorylation state of typically 2–4 different protein markers such as the cell surface protein Epithelial Cell Adhesion Molecule (EpCAM) and total or phosphorylated Erk1/2 and Akt proteins—the latter representing the key protein of the PI3K/Akt/mTOR pathway—in a direct fluorescence immunoassay with the proven specific RPPA-tested primary antibodies. The remaining arrays were used as control arrays to address potential auto-fluorescence and blank signal contributions in the absence of specific primary antibodies.

#### Data analysis of single cell lysate spot signals

The mean signals of the printed spots were analyzed using standard Image J software routines (version 1.50f, NIH, USA) and a flexible array of analysis spot circles. To account for variations in spot size due to capillary-to-capillary or single cell-to-single cell lysate variations, spot signals were analyzed as a mean signal density, with the spot area set constant for all printed spots at a diameter of approximately 150 μm to exclude background pixels. This was well comparable to the 160 μm analysis spot diameter applied in standard RPPA [31] and ensured reproducibility of the quantified assay signals from the technical spot replicates with mean coefficients of variation (CVs) well below 10% with a range between 4% and 7%. No significant variation of the mean signals from different arrays was observed. These observations were indicative of the robust printing and complete single-cell lysis processes. Unless otherwise stated, mean spot signals of the primary antibody and the blank assays were averaged to the mean values of the technical replicates (*n* = 2–3). Primary assay means were then corrected for the respective mean blank assay contributions in the absence of primary antibody, but under otherwise comparable conditions and given as Mean Fluorescence Intensity (MFI) values.

### Protein analysis in single cells

#### EpCAM expression

The cell surface protein EpCAM represents the main target to detect and isolate CTCs in MBC. We, therefore, decided to measure EpCAM expression levels first on single BC cell line cells to verify the workflow’s performance and to prove the sensitivity of ZeptoChip’s readout. For this purpose, single EpCAM-positive MCF-7 and EpCAM-low/negative MDA-MB-231 cells were isolated and processed as described above.

The lysates of two separately processed pairs of single MCF-7 and MDA-MB-231 cultured cells (cell1, cell2) were printed in technical replicates (2 spots; *n* = 2) onto ZeptoChip replica arrays. On a separate replica array, only a secondary antibody was applied as a blank assay control.

**Fig. 3:**
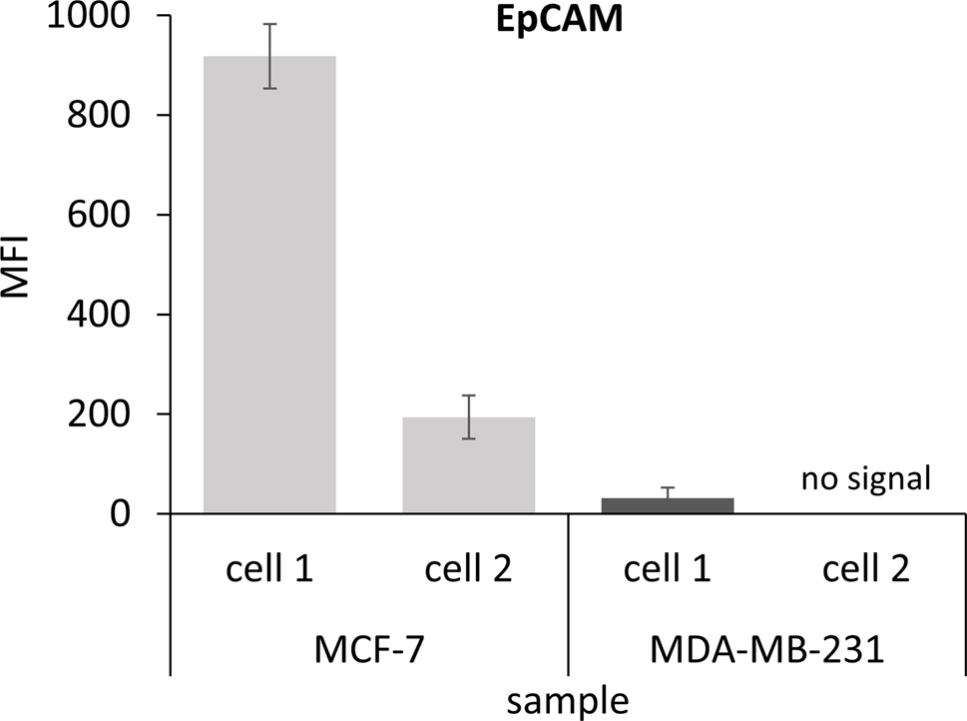
EpCAM protein expression signals measured from single cell preparations using ZeptoChip. Quantification of fluorescence signals depicted from two MCF-7 and two MDA-MB-231 single cell lysate samples, respectively. The mean fluorescence signals (MFI) of both MCF-7 single cells were significantly higher than those of both MDA-MB-231 cells (p-value = 0.0435). Error bars indicate the standard deviations of the mean technical replicate spot signals (*n* = 3)

After measuring the signal intensities with the ZeptoReader, the fluorescence images with the longest exposure time (4s) below pixel saturation (16 bit) were analyzed and single cell lysate spot signals were quantified as blank-corrected mean spot signals averaged over the technical replicates. As outlined in Fig. 3, the EpCAM MFI signals for both MCF-7 single cell preparations were detected well above blank, and were—as expected—significantly higher than for the EpCAM-low expressing MDA-MB-231 cells. The MDA-MB-231 cell 1 revealed very low, but still detectable residual EpCAM expression levels, for cell 2 no signal was detected. Moreover, these first workflow experiments yielded < 10% mean signal CVs ranging from 3 to 8% of the printed technical spot replicates, and hence indicated a good spot-to-spot reproducibility of cellular EpCAM protein detection (see also low error bars in Fig. 3 indicating standard deviations). Additionally, the EpCAM signals of two cells randomly picked from the same cell line (MCF-7, MDA-MB-231) were considerably different pointing towards well-known cell-to-cell heterogeneity. Nonetheless, such single cell protein variability and environmental changes seem now addressable with the presented method.

#### Measuring phosphorylated Akt in single tumor cell line cells upon Capivasertib treatment

**Fig. 4:**
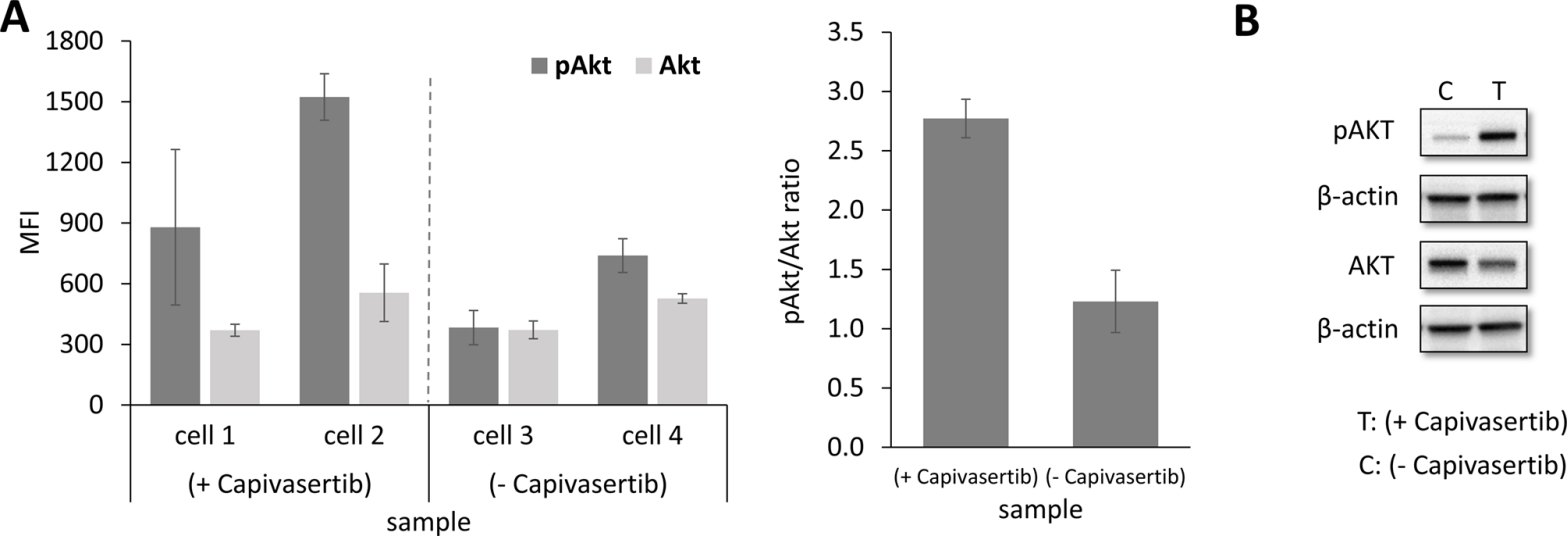
Expression signals of Akt and pAkt (Ser473) in treated single MCF-7 cells (description see main text)

In the next step, we tested if our workflow can be used to measure in parallel expression level and its phosphorylation status of a signal protein of interest. Since the PI3K/Akt signaling pathway represents a key pathway not only in BC development but also in its therapy resistance, we selected the Akt protein and its functional form phosphorylated at serine 473 (Akt-P-Ser473), in short pAkt, as key analytes. Phosphorylated Akt was also good to proof for sensitivity, since especially this phosphorylated analyte (and also others) is known for its relative low abundance and hence a challenge of detection. MCF-7 cells were treated with 5 µM capivasertib for 24 h. Capivasertib is an Akt1-specific inhibitor and is known to lead to elevated pAkt levels, due to its ATP-competitive mechanism of action and accumulation of inactivated hyper-phosphorylated Akt [36]. The increased pAkt levels were verified by Western Blot analysis of lysates from bulk MCF-7 cells (Fig. 4B). From the same cell culture used for Western Blot analysis, capivasertib-treated single MCF-7 cells (MCF-7/treated, 3 single cells) and untreated control cells (MCF-7/untreated, 2 single cells) were micromanipulated and processed according to the established workflow. Four technical replicates per single cell lysate were printed for each replica array. The arrays were incubated with primary antibodies against Akt and pAkt, followed by the anti-species Alexa Fluor™ 647 conjugated secondary antibody and fluorescence detection with the ZeptoReader (Fig. 4). One array was used as blank control.

Blank-corrected MFI fluorescence signals for pAkt in single MCF-7 cells were significantly higher compared to the total Akt protein (see Fig. 4). Treatment-to-Control Ratio (TCR) for pAkt as calculated from the respective single cell MFI mean values showed clear and significant up-regulation (2.4 fold, *p* = 0.0047) in MCF-7-treated over MCF-7-non-treated single cells (Fig. 4A left). In contrast, the TCR of total Akt protein remained almost unchanged (1.1), as expected from capivasertib’s exclusive effect only on the functional phosphorylation of Akt (see Fig. 4A left). Consequently, the mean TCR of the Akt-normalized pAkt/Akt ratios was then also more than 2-fold significantly higher (2.8 fold, *p* = 0.0012) in the MCF-7/treated over the MCF-7/non-treated cells (see Fig. 4A right). The observed single cell results have been verified by co-performed Western Blot analysis with bulk cell pellet lysate preparations (> 2 × 10^6^ cells starting material) from respective same treated and non-treated MCF-7 cell line cultures, (with about 3-fold TCR of pAkt/Akt (see Fig. 4B).

In summary, the data obtained with ZeptoChip detection and true single cell lysates were well in accordance with western blot analysis measured from more than 2 × 10^6^-fold starting cell material and supported that the established workflow is highly sensitive and able to measure both the expression and functional (phosphorylation) status of signaling protein markers in parallel from single cells and in a multiplex format.

### Application of the single cell protein analysis workflow to CTCs

In order to adapt the workflow to CTCs, we decided to use fresh frozen aliquots of DLA-products for mainly two reasons [34]. First, the cells are not treated with a fixative such as paraformaldehyde, keeping the CTCs viable and avoiding negative effects hampering cell processing and the measurement of proteins and their phosphorylation levels. Second, since one aliquot is always inspected with the CellSearch® System, both CTC numbers and their expected quality were known.

#### Detection and processing of single CTCs

DLA aliquots were processed with the CellSearch® profile kit and the CellSearch® system to capture CTCs. Since it is essential to prevent the permeabilization of the cell membrane to avoid the loss of intracellular proteins, the CTCs were identified with a mixture of antibodies targeting only surface proteins [37]. Selection of the used antibodies, their type as well as applied detection labels were carefully coordinated with the RPPA detection routines. The labeled cells were scanned with the CellCelector™ automated micromanipulator (Sartorius, Jena), and single CTCs were isolated and lysed, as described before for the single cancer cell preparations.

The isolated single CTC lysates were printed onto the ZeptoChip slide in the array formats as described before.

#### Measuring phosphorylated Akt and Erk1/2 in CTCs derived from a MBC patient

Since we aimed to measure protein expression level and in parallel its functional phosphorylation status in single CTCs, we selected a cryopreserved and CTC-positive DLA product obtained from an MBC patient (patient 1, see additional file 2). Prior DNA sequencing analysis had confirmed that approx. 80% of her CTCs harbored the activating Akt1(E17K) mutation (see additional file 3) [38].

Although this mutation should result in the phosphorylation of the Akt protein at serine 473 [39], we did not know at which level and frequency. This sparked us to also measure the phosphorylation level of Erk1/2 protein. This protein is a key regulator of the MAPKinase signaling pathway for cellular growth, often interacting directly with the PI3K/Akt pathway in a cross-compensatory manner. For total Erk1/2 and phosphorylated Erk1/2 (Erk1/2-P-Thr202/Tyr204) proteins, well validated and measurable assays were available (since these protein forms are often presented at higher abundance/active state than e.g., pAkt), as part of our established list of RPPA assays (700 + antibodies). For this experiment, we also included—apart from the single CTC samples - lysates of single patient-matched WBCs, since in contrast to treated or untreated cell lines we do not dispose of ‘unstimulated’ CTCs from the same patient. Furthermore, we included validated RPPA standard lysates, which we regularly apply in routine RPPA protein profiling studies, as quality control (QC) for array printing and assay performance. These standard QC lysates had been prepared upfront and characterized from large bulk amounts of MCF-7 tumor cell line cultures (> 10 × 10^6^ cells). These standard samples came as aliquoted pairs of treated and control (non-treated) MCF-7 lysates (kindly provided by NMI TT, Reutlingen, Germany) with pre-confirmed Akt and pAkt (and other protein) levels, and were co-printed onto the replica arrays of this experiment, at a concentration representing also single cell equivalent total protein material per spot, together with the true single CTC and WBC sample lysates.

**Fig. 5:**
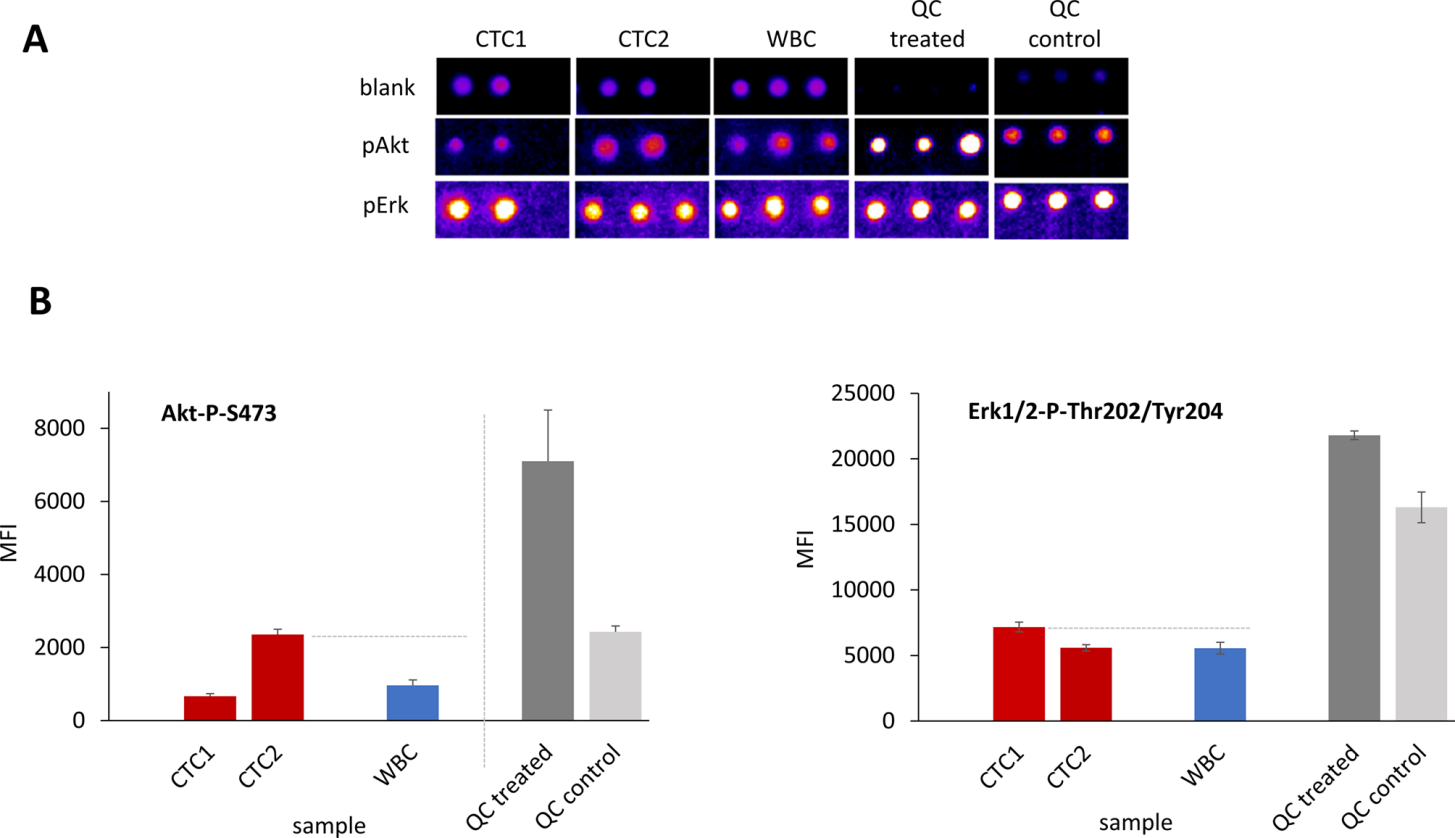
Evaluation of phosphorylated Akt and Erk1/2 proteins in single CTCs derived from a MBC patient. (**A**) False color clippings of RPPA Zeptosens fluorescence array images (exposure 4s) for analysis of pAkt and pErk1/2 protein levels in CTC and WBC single cells from a MBC patient, and comparison to bulk cell QC (MCF) controls (3 replicate spots printed per sample). (**B**) Mean fluorescence signals (MFI) as quantified by ImageJ: elevated pAkt and pErk1/2 levels in CTCs compared to WBCs in line with QC lysates; pErk1/2 abundancy signal 3–11 times higher than pAkt as evident from different MFI scales

Two different CTCs were isolated from a thawed DLA product and processed as described above (Fig. 5A) (see additional file 4). All CTCs, WBCs, and standard cell lysates were printed in technical replicates into ZeptoChip replica arrays, with single cell lysate volumes sufficient to determine the multiple Akt-P-Ser473 (pAkt), Erk1/2-P-Thr202/Tyr204 (pErk) protein and blank assay signals, respectively. The quality of the printed arrays is illustrated in Fig. 5 (top) with clippings of the fluorescence image recordings. The lysate print achieved homogeneous spot morphology, uniform spot diameters and a good signal reproducibility of the printed spots, evident from a low mean coefficient of variation (CV = 6%) of the sample MFIs quantified by the ImageJ software and averaged overall the different samples printed on the chip.

The blank-corrected mean fluorescence intensities (MFIs) showed more than two-fold difference among the different CTCs and were clearly more pronounced for pAkt than for pErk1/2 levels (see Fig. 5B). One of the two printed single CTC lysates (CTC2) showed a clear and significantly higher pAkt level compared to the patient-matched WBC (2.4-fold, *p* = 0.02), whereas the pErk1/2 levels (CTC1 and CTC2) were almost comparable (1.2-fold, *p* = 0.02). Notably, the pErk1/2 abundancy signals were 3–11 times higher than for pAkt as evident from the different absolute MFI scales (Fig. 5B). Significantly elevated pAkt and pErk1/2 levels observed in the treated standard QC lysates were well in agreement with the measured enhanced signals in the CTCs. Also, the absolute signal levels of the co-printed standard QC controls and the single CTC lysate MFIs were well in a comparable order range, even though they were prepared according to completely different protocols (one prepared from bulk cells and the other from only single cell amounts). Besides, the TCRs of the standard QC lysates were at well comparable values and quality when printed and measured (i) in a routine RPPA setting (> 10^6^ cells lysed at mL volumes) and (ii) in the new, true single cell lysate workflow (single cells lysed at few nL volumes).

These data demonstrate that the new workflow can measure multiple proteins expressed with low and high abundancies from true single cell sample preparations.

#### Measuring phosphorylated Akt protein in single CTCs derived from two MBC patients

**Fig. 6:**
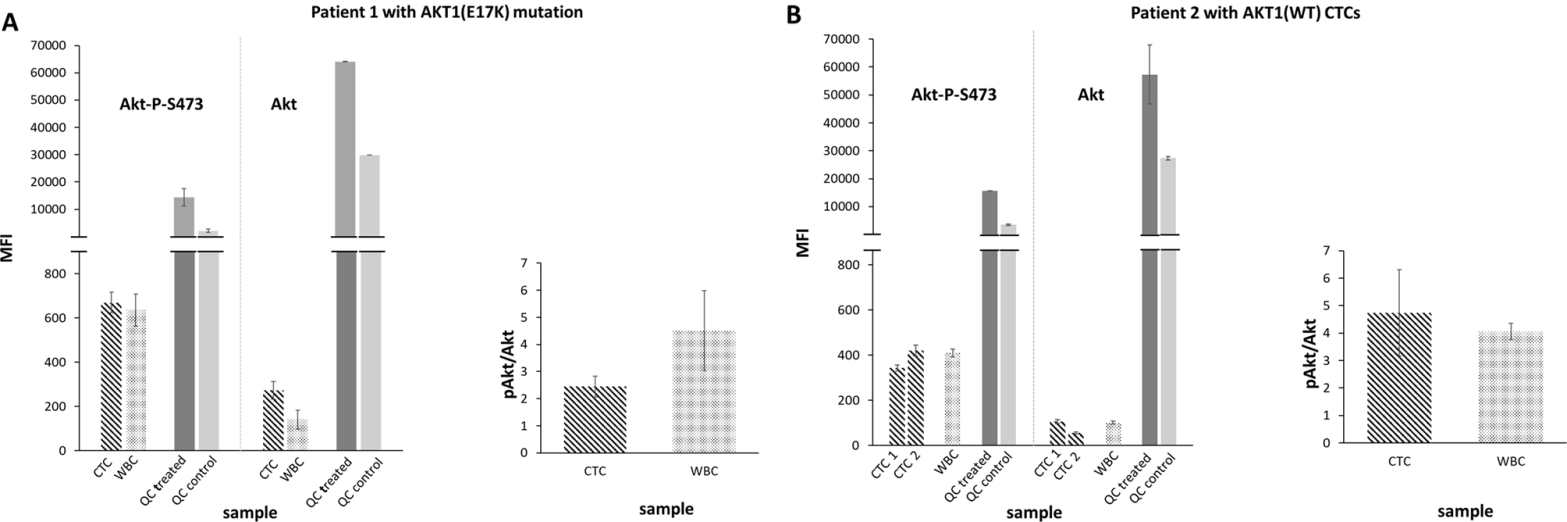
Measuring pAkt to Akt protein in single CTCs derived from two index MBC patients. (**A**) (left) RPPA Zeptosens analysis of pAkt and Akt protein signals (MFI) in single CTC and WBC samples derived from index patient 1 harboring an Akt1(E17K) mutation, in addition to co-printed quality control samples (QC treated, QC control); (right) pAkt/Akt mean signal ratios of respective patient 1 single CTC and WBC samples. (**B**) (left) RPPA Zeptosens analysis of pAkt and Akt protein signals (MFI) in single CTCs and WBCs from index patient 2 (wild type); (right) pAkt/Akt mean signal ratios of respective patient 2 single CTC and WBC samples

As a next step, we aimed to investigate the potential of the workflow in measuring expression and activation signals in CTCs derived from patient samples, both with and without the Akt1(E17K) mutation. To achieve this purpose, cryopreserved and CTC-positive DLA products were obtained from patient 1 [Akt1(E17K)] and another MBC patient (patient 2) with CTCs of wild-type AKT1 genotype (Supplement Seq Data). Single CTCs and WBCs were picked with the CellCelector™ and processed as described above (see Fig. 6A and B for results). The printed single cell CTC lysates of the two patients, co-printed patient-matched WBC lysates, and standard QC lysates were investigated, this time with a focus on pAkt and total Akt protein levels. As indicated by the low standard deviations and the QC standard lysate signals (Fig. 6), the quality of the printed arrays and the reproducibility of the replica spot MFI assay signals were again good and successfully attained. The CVs of mean replica spot MFI assay signals averaged over all printed CTC and WBC single cell lysates of patient 1, were 3% for the pAkt and for the total Akt. For the CTC and WBC single cell lysates of patient 2, similar low mean CVs were achieved (5% for pAkt and 3% for total Akt, respectively). With these numbers, print and assay signal CVs of the application were well comparable with those reported for standard RPPA applications (see [31] for numbers). The MFI signals and TCR of the co-printed standard QC lysates for both pAkt and Akt confirmed the correct readout process for both pAkt and total Akt. In the CTC single cells from patient 2 with AKT1 wildtype CTCs, both the Akt and pAkt average absolute signals were slightly below than in the patient-matched WBC (0.9-fold for pAkt, 0.8-fold for Akt), see Fig. 6B left. This resulted in about comparable pAkt/Akt mean signal ratios (5.4 fold for CTC, 4.1 fold for WBC), see Fig. 5B right. The pAkt/Akt signal ratios indicate that also pAkt/Akt levels in these two cell types were almost comparable. In CTCs isolated from patient 1 with AKT1 mutated CTCs, slightly higher absolute pAkt (1.1-fold) and almost double-fold Akt signals (1.9-fold) were observed compared to the respective WBCs (Fig. 6A left). This resulted in reduced pAkt/Akt signal ratios in the CTCs (mean 2.4-fold) compared to the WBCs (mean 4.5-fold, comparable to the respective wild-type ratio), see Fig. 6A right, mainly due to increased relative total Akt in the mutated compared to wild-type CTCs.

Assessing also the good reproducibility of the quality control signal intensities in both chips (mean CV < 10%), in the patient with harboring CTCs with Akt1(E17K) mutation, the absolute MFIs of the CTCs lysates in the pAkt assay were clearly higher (1.8-fold) than those of the CTC lysates from the patient with AKT1(WT) CTCs (670 ± 47 for mutated versus 382 ± 39 for wildtype), and were even higher (3.4 fold) in the total Akt assay (274 ± 39 for mutated versus 81 ± 26 for wild-type). Although we do not know the AKT1 genotype of each CTC investigated, this observation suggests that the workflow has the capacity to measure a higher signal intensity for pAkt in CTCs obtained from the MBC patient with Akt1(E17K) mutated CTCs compared CTCs from the MBC with AKT1(WT) CTCs.

In summary and in line with our quality control measures, these data demonstrate that the ZeptoCTC workflow is robust and able to measure target proteins in single CTCs. Furthermore, the possibility to process up to 6 replica arrays/assays in a single chip run with a number of different single cell lysate preparations, opens the window to analyse several key pathway protein markers in parallel in a flexible (RPPA like) multiple assay and sample setting format.

## Discussion

Rare cells, such as circulating tumor cells (CTCs) from blood-based liquid biopsies and stem cells, offer unique opportunities for multi-omic analysis, though protein analysis remains challenging due to the lack of amplification mechanisms. To address this, we developed the ZeptoCTC workflow—an ultra-sensitive tool for single-cell protein analysis, initially focusing on CTCs. This workflow efficiently enriches, detects, and isolates viable CTCs while differentiating them from apoptotic cells, ensuring high-integrity protein analysis. ZeptoCTC excels at measuring ultra-low protein abundances and their post-translational modifications in individual CTCs. Our proof-of-concept establishes a sophisticated, miniaturized approach, integrating advanced methodologies for single-cell detection, isolation, and high-sensitivity protein analysis. This is particularly valuable for scenarios with very limited cell populations, such as CTCs in cancer patients’ blood.

### Need for functional testing of cancer gene variants

Molecular analysis of CTCs is essential for identifying and monitoring targeted treatments, especially when tissue biopsies are impractical. However, genetic changes alone often do not fully capture the activation status of protein products, such as phosphorylation levels or signaling pathway activation. For instance, breast cancer cell lines with PIK3CA hotspot mutations show varied responses to PI3K inhibitors, and additional mutations affecting PI3K activity and tumor growth have been identified [11, 18, 40–44]. Similarly, mutations in the AKT1 gene affect oncogenic activity [45]. These examples highlight the need for to combine DNA mutation data with single-cell protein analysis to provide crucial insights for precise therapy selection and accurate disease state determination. However, in the realm of single-cell proteomics analysis of CTCs, only few studies have utilized real single CTCs from patient blood to detect low-abundance proteomic content [46, 47]. One study employed a combination of FACS and nanoPOTS platform with automated LC-MS systems to identify 252 proteins, but did not analyze real single CTCs [48].

### Limited utility of currently available protein technologies for single cell protein analysis

The investigation of proteins—and, more importantly, their functional activation—at low-abundance levels and on the single-cell level is often constrained by the limited detection sensitivities of current protein technologies or their requirement for high starting cell numbers [20]. Protein analysis approaches, including highly multiplexed methods such as flow cytometry [21], mass spectrometry [22], and mass cytometry—CyTOF [23], as well as single-cell absolute protein quantification methods using adapted immunoassay-based Western Blot [24] and ELISA [25] often involve sophisticated, miniaturized versions of mature technologies. These methods achieve high sensitivities by detecting very dilute (fg/ml) analyte concentrations [26] or by enhancing analyte specificity with nucleic-acid (NA)-based recognition elements [27]. However, they are generally not suitable for analyzing single CTCs, as they are not optimized for single-cell input and suffer from analyte loss during sample processing [28]. In contrast, ZeptoCTC represents an extremely sensitive protein analysis method that integrates seamlessly into an established and robust workflow for single CTC or other rare cell detection, isolation, and preparation. Although not yet demonstrated, a significant advantage of ZeptoCTC over many of the aforementioned technologies is that it allows to separate the cellular nucleus from the cytoplasm using a mild lysis buffer, enabling parallel single-cell DNA analysis, such as detecting AKT1 mutations and related Akt signaling on the protein level, which represents a clear limitation of the here presented work (see below).

### Key results

The key results of our study highlight the ZeptoCTC workflow’s outstanding ability to produce precise and insightful multi-protein expression data at the single-cell level using a miniaturized, highly sensitive RPPA fluorescence read-out system. This workflow delivers excellent signal-to-noise ratios and minimal signal variations (high precision), achieving coefficients of variation (CVs) below 10% among technical replicates and demonstrating low variability between processed chips. This consistency is evident from the reproducible measurements of pAkt and Akt signals obtained from co-printed, validated RPPA control lysates.

Due to this precise performance of the miniaturized workflow, we argue that the observed signal variabilities observed between single cells—also of the same cell line—are pointing toward well-known cell-to-cell heterogeneity, a phenomenon that has been previously noted in other single cell experiments [49–51]. For instance, EpCAM expression levels in two MCF-7 cells were highly different, matching observations we have made previously suggesting a continuum of EpCAM-positivity in MCF-7 cells [37]. Similarly, varying levels of Akt and pAkt in single MCF-7 cells treated with Capivasertib suggest diverse drug responses within individual cells.

Our workflow also demonstrates high sensitivity by detecting low-abundance proteins, including Akt in both its total and phosphorylated forms (Akt-P-S473 known to be very low abundant), from just a 1/20 volume of the complete single-cell lysate per spot. This capability facilitates the design of assays that can analyze additional proteins and critical signaling pathways, such as those involved in the Her2/PI3K/Akt/mTOR signaling pathway in breast cancer. Future work will focus on optimizing these assays through further titration experiments to expand their scope and enhance their applicability for comprehensive, personalized cancer research and therapy.

### The ZeptoCTC workflow’s advantages

#### Integration of sensitive technologies

The ZeptoCTC workflow stands out by integrating advanced techniques for cell identification, processing, and protein analysis. It starts with the precise detection and isolation of individual tumor cells using the FDA-approved CellSearch® system. This pre-analytical process well defines upfront the CTC population and reduces background interference and maintains cell integrity. This is followed by detailed downstream protein analysis with the Zeptosens RPPA.

The workflow combines micromanipulation, lysing, and spotting of isolated individual CTCs onto a surface or into a tube, ensuring minimal loss of cells and lysate and allowing for immediate printing. Further, ZeptoCTC is adaptable, integrating various enrichment methods or antibody cocktails to target and isolate different CTC subtypes. A key feature is the live camera embedded in the CellCelector™ device, which provides real-time imaging to refine each step—from isolation to lysing and printing—thereby enhancing overall accuracy and efficacy.

#### Detection of low abundance proteins

ZeptoCTC’s success in detecting low-abundance proteins is due to its use of highly specific antibodies and advanced planar waveguide-based fluorescence detection, allowing it to achieve a detection limit in the zeptomole range - roughly 1000 protein molecules (~ 100 attogram) per spot from a total of approximately 10^9^ protein molecules (~ 100 pg) measured by the ZeptoREADER. This exceptional sensitivity is evident from our study, where MCF-7 single-cell lysate droplets exhibited higher fluorescence intensity for EpCAM compared to MDA-MB-231 lysates. The workflow’s high sensitivity is further enhanced by adjusting the sample lysis volume to 40 nL per single cell and using only 2 nL per spot on the ZeptoChip, which results in lower lysate consumption and improved sensitivity relative to methods like SCoPE-MS [52] and OAD and nanoPOTs [48, 53], which require larger sample volumes.

ZeptoCTC demonstrates robustness and good precision by consuming up to 20 spots per single-cell lysate sample prepatation (40 nL), with each spot allowing measurement of up to 6 different protein assays (e.g. here, Erk, pErk, Akt, pAkt plus 2 controls). This setup includes 3–4 technical replicates per condition across 6 sample array replicates, showing strong precision in signal generation. The platform can currently measure up to 10–20 proteins per chip (at a lower technical replicate number), with the potential to double this number using two-color fluorescence.

While ZeptoCTC is not designed for broad proteome identification, it excels in validating candidate proteins, their modifications, and drug responses at the single-cell level. This is particularly valuable for targeted pathway analysis, such as the PI3K/Akt/mTOR pathway in luminal breast cancer, where the precise measurement of pathway activation or inhibition can guide personalized therapy. Thus, ZeptoCTC is envisioned as a powerful tool for targeted protein analysis, providing critical insights into the activation status of key signaling pathways essential for personalized cancer treatment.

#### Single-cell protein analysis

ZeptoCTC’s focus on lysing and analyzing single cells offers a significant advantage over traditional bulk proteome digestion methods, especially for CTCs in patient blood. Bulk lysate approaches can introduce technical and biological confounding factors, as they are designed for larger cell populations and may not address the nuanced requirements of single-cell analyses. Our approach, demonstrated with a reference quality control lysate, effectively highlights differences between treated and control groups, revealing higher mean fluorescence signals compared to single CTCs and white blood cells. This underscores ZeptoCTC’s precision and sensitivity in analyzing rare and heterogeneous single-cell samples.

### Limitations and outlook

Despite the ZeptoCTC workflow’s impressive precision and sensitivity, several limitations warrant attention. First, the data is based on a limited sample set, demonstrating low variance among replicates and showcasing the workflow’s robustness, but a larger clinical study is necessary for broader validation and generalizability. The current study should be viewed as a proof-of-concept / clinical applicability to establish a sophisticated, further miniaturized, and integrated workflow. At this stage, we believe that adding 1, 2, or even 5 more patient samples will not significantly enhance the message we aim to convey. Second, the process is also time-consuming and lacks full automation, which impacts ease of use, flexibility, and throughput; thus, future advancements should focus on enhancing automation and streamlining the workflow. Third, software analysis faces challenges such as irregular spot morphologies, variations in spot size, and blank corrections. Integrating alternative printing techniques and advanced software solutions could improve performance and accuracy. Fourth, the setup’s current limitations in analyzing multiple CTCs and proteins simultaneously suggest that future improvements could involve chips with more protein arrays, enabling comprehensive analysis of key signaling pathways in a single run. This would be crucial for advancing personalized therapy and studying other rare single cells. Sixth, the inability to concurrently perform DNA and protein analysis restricts our capacity to confirm genetic mutations in CTCs analyzed for protein expression. Developing methods to separate the cell nucleus from the cytoplasm or the use of antibodies specific for targeted mutations, would significantly enhance the exploration of the interplay between genetic mutations and protein expression in CTCs. Overall, while ZeptoCTC marks a significant advancement in single-cell protein analysis, ongoing development and optimization are essential to overcome these limitations and fully realize its potential in precision medicine.

## Conclusions

The ZeptoCTC workflow represents a significant advance in cancer research, particularly in the realm of protein analysis on the single cell level. This innovative approach offers new insights into the relationship between genetic mutations, protein signaling and drug actions in all types of rare cells like CTCs, enhancing our understanding of tumor heterogeneity and the development of targeted therapies. Key findings include the precise detection of low-abundance proteins and the ability to manage cell-to-cell variability, making it a valuable tool for personalized medicine. While there are limitations such as the need for further automation and the challenge of simultaneous DNA and protein analysis, this technology opens new avenues for cancer diagnosis, treatment, and understanding, marking a substantial step forward in bridging laboratory research with clinical applications.

## Electronic supplementary material

Below is the link to the electronic supplementary material.

Supplementary Material 1

Supplementary Material 2

Supplementary Material 3

Supplementary Material 4

## Data Availability

Data and materials will be provided by the corresponding author upon request.

## Abbreviations

Akt: Protein kinase B
BSA: Bovine serum albumin
BC: Breast Cancer
CLB1: Cell Lysis Buffer
CSBL1: Cell Spotting Buffer Lysate
CTC: Circulating tumor cell
CyTOF: Cytometry by the time of flight
DLA: Diagnostic leukapheresis
DPBS: Dulbecco’s Phosphate Buffered Saline
EpCAM: Epithelial Cell Adhesion Molecule
Erk1/2: Mitogen-activated protein kinase
ESR1: Estrogen Receptor 1
IC50: Half-maximal inhibitory concentration
MBC: Metastatic breast cancer
MFI: Mean Fluorescence Intensity
mTOR: Mammalian Target of Rapamycin
NAs: Nucleic acids
pAkt: Akt-P-Ser473 or phosphorylated Akt
pErk: Erk1/2-P-Thr202/Tyr204 or phosphorylated Erk1/2
PI3K: Phosphoinositide-3-kinase
PIK3CA: Phosphatidylinositol-4,5-Bisphosphate 3-Kinase Catalytic Subunit Alpha
PT: Primary tumor
RPPA: Reverse Phase Protein Arrays
TCR: Treatment-to-Control Ratio
WBC: White Blood Cell
w/v: Weight per Volume
