# Supplementary material for: Miniaturized protein profiling permits targeted signaling pathway analysis in individual circulating tumor cells to improve personalized treatment
Source: J Transl Med. 2024 Sep 20;22:848. doi: 10.1186/s12967-024-05616-7 (PMC11414235; doi:10.1186/s12967-024-05616-7)
Supplement: Supplementary file 1 — Supplementary Material 1 [file 12967_2024_5616_MOESM1_ESM.docx]

**Supplemental Materials**

**Additional file 2. Supplemental Table 1. Clinicopathological characteristics of the index metastatic breast cancer patients.**

| Patient ID | Age | Tumor size* | Nodal status* | Metastasis status* | Histology | Grading | Molecular Subtype | Lines of therapy in metastatic situation |
| --- | --- | --- | --- | --- | --- | --- | --- | --- |
|  |  |  |  |  |  |  |  |  |
| AKT1 mutated | 74 | 2 | 0 | 0 | Invasive lobular | 2 | Luminal | 2 |
| Not AKT1 mutated | 46 | 1 | 1 | 0 | NST | na | Luminal | 1 |

*At the time of diagnosis; na: Not analyzed


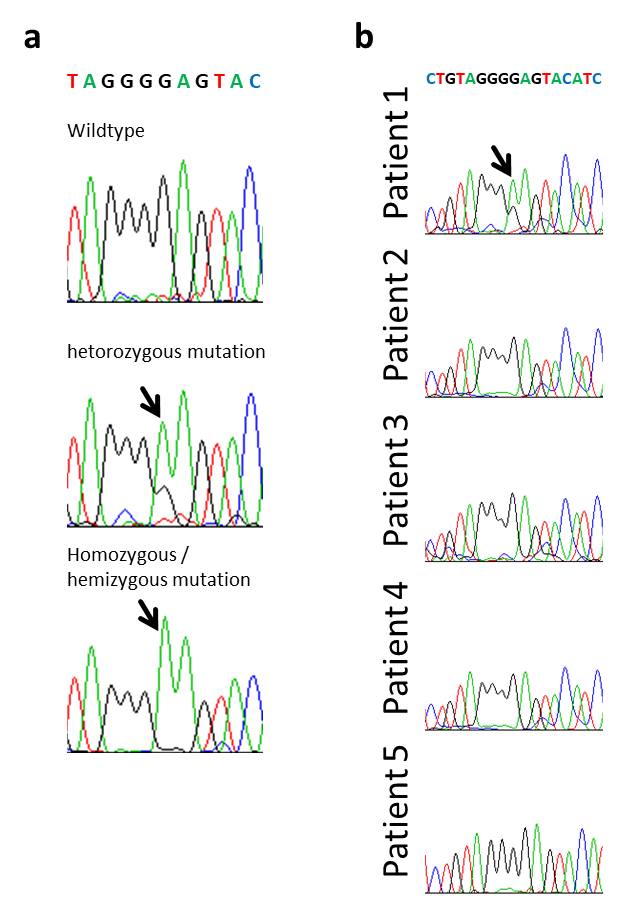


A

B


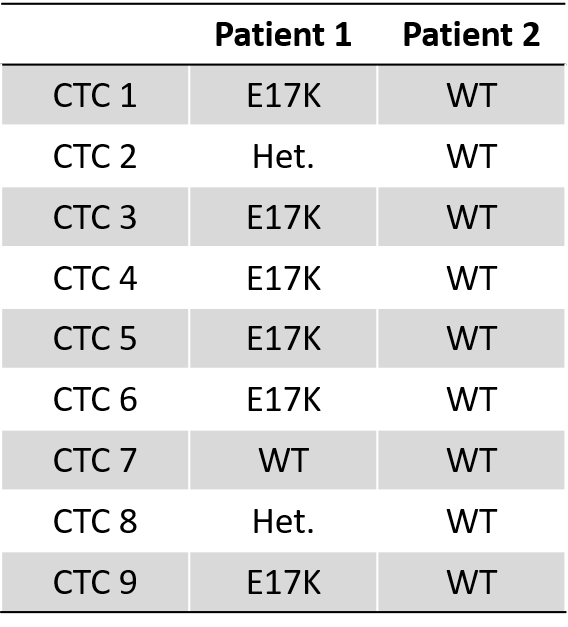


**Additional file 3. Supplemental Figure 1. Mutation analysis of the AKT1 E17K hotspot in two index breast cancer patients.**

(A) References for E17 hotspot mutation in AKT1. The Analysis was performed by Sanger Sequencing. (B) The E17 hotspot region of AKT1 was analyzed on WGA products from 9 CTCs of each patient. WT, wild-type; Het., heterozygous


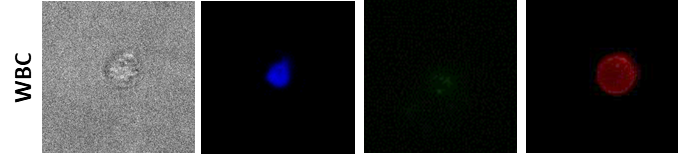

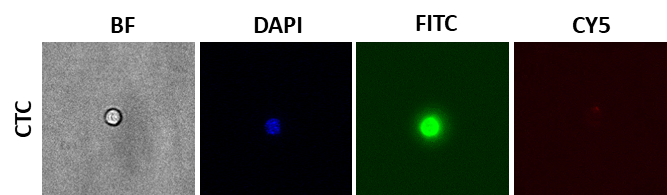


50 µM

25 µM

**Additional file 4. Supplemental Figure 2. Fluorescence microscopy of two CTC and WBC from a breast cancer patient using CellCelector^TM^ stage microscopy.**

In Panel A (CTC), CTC exhibits surface markers' signals for cytokeratin (FITC) and nuclei (DAPI) while demonstrating a negative signal for CD45 (CY5). The scale bar in Panel A measures 50 µM. In Panel B (WBC), WBC presents nuclei (DAPI) and CD45 (CY5) signals, with a negative signal for cytokeratin (FITC). The scale bar in Panel B measures 25 µM.
